# Compliment to Dr. J. P. White, Prof. of Obstetrics and Diseases of Women and Children, Med. Dept. University of Buffalo

**Published:** 1847-07

**Authors:** 


*Compliment to Dr. J. P. White, Prof. of Obstetrics and Diseases of Women and Children, Med. Dept. University of Buffalo.*

On the conclusion of the lectures by Prof. White, in the Medical Department of the University of Buffalo, at its late session, the class appointed a committee to express their high sense of his professional attainments,as exemplified in his valuable course of instruction; to tender their congratulations on the success attending his first professorial labors, and to acknowledge their obligation for his kindness and courtesy in all their official and social relations during the session.

